# Early postoperative serum S100β levels predict ongoing brain damage after meningioma surgery: a prospective observational study

**DOI:** 10.1186/cc5058

**Published:** 2006-10-04

**Authors:** Sharon Einav, Yigal Shoshan, Haim Ovadia, Idit Matot, Moshe Hersch, Eyal Itshayek

**Affiliations:** 1 General Intensive Care Unit, Shaare Zedek Medical Centre (affiliated with the Faculty of Health Sciences of the Ben-Gurion University), PO Box 3235, Jerusalem 91031, Israel; 2 Department of Neurosurgery, Hadassah-Hebrew University Medical Centre, POB 12000, Jerusalem 91120, Israel; 3 Department of Neurology, Agnes Ginges Centre for Human Neurogenetics, Hadassah-Hebrew University Medical Centre, POB 12000, Jerusalem 91120, Israel; 4 Department of Anaesthesia and Intensive Care Medicine, Hadassah-Hebrew University Medical Centre, POB 12000, Jerusalem 91120, Israel

## Abstract

**Introduction:**

Elevated serum levels of S100β, an astrocyte-derived protein, correlate with unfavourable neurological outcomes following cardiac surgery, neurotrauma, and resuscitation. This study evaluated whether pre-/postoperative serum S100β levels correlate with unfavourable clinical and radiological findings in patients undergoing elective meningioma resection.

**Methods:**

In 52 consecutive patients admitted for meningioma surgery, serum S100β levels were determined upon admission and immediately, 24 hours, and 48 hours after surgery. All patients underwent complete pre- and postoperative neurological examination and mini-mental state examination. Radiological evaluation included preoperative magnetic resonance imaging (MRI) and postoperative computed tomography. Tumour volume, brain edema, and bleeding volume were calculated using BrainSCAN™ software.

**Results:**

Preoperative S100β levels did not correlate with the tumour characteristics demonstrated by preoperative MRI (for example, tumour volume, edema volume, ventricular asymmetry, and/or midline shift). Preoperative serum S100β levels (0.065 ± 0.040 μg/l) were significantly lower than the levels measured immediately (0.138 ± 0.081 μg/l), 24 hours (0.142 ± 0.084 μg/l), and 48 hours (0.155 ± 0.119 μg/l) postoperatively (*p *< 0.0001). Significantly greater postcraniotomy S100β levels were observed with prolonged surgery (*p *= 0.039), deterioration in the mini-mental state examination (*p *= 0.005, 0.011, and 0.036 for pre versus immediate, 24 hours, and 48 hours postsurgery, respectively), and with postoperative brain computed tomography evidence of brain injury; bleeding was associated with higher serum S100β levels at 24 and 48 hours after surgery (*p *= 0.046, 95% confidence interval [CI] -0.095 to -0.001 and *p *= 0.034, 95% CI -0.142 to -0.006, respectively) as was the presence of midline shift (*p *= 0.005, 95% CI -0.136 to -0.025 and *p *= 0.006, 95% CI -0.186 to -0.032, respectively). Edema was associated with higher serum S100β levels immediately (*p *= 0.022, 95% CI -0.092 to -0.007) and at 48 hours after surgery (*p *= 0.017, 95% CI -0.142 to -0.026). The degree of elevation in S100β levels at 24 and 48 hours after surgery also correlated with the severity of midline shift and edema.

**Conclusion:**

In patients with meningioma, serum S100β levels perform poorly as an indicator of tumour characteristics but may suggest ongoing postcraniotomy injury. Serum S100β levels may serve as a potentially useful early marker of postcraniotomy brain damage in patients undergoing elective meningioma resection.

## Introduction

S100β is a calcium-binding protein usually found in astrocytes. Its biological half-life is approximately 30 minutes [1]; hence, persistently increased levels of S100β indicate continuous release of this protein from damaged tissue. Elevated serum levels of S100β have been reported to correlate with neurological deterioration after cardiac surgery [2,3] and with poor likelihood of survival after hypoxia [4]. Serum protein S100β is also a recognised marker of traumatic brain injury [5-7] and blood-brain barrier dysfunction in the absence of apparent brain injury [8]. Few studies have evaluated S100β after surgical insult to the central nervous system (CNS); after aneurysm surgery [9] and operative decompression of cord metastases [10], increased serum S100β values were reported to correlate with poor neurological outcome.

Slow-growing supratentorial brain tumours such as meningiomas may cause damage to adjacent neural tissue despite their non-neural origin. Surgical access and excision of these extra-axial tumours are generally less traumatic than in less-accessible brain tumours or tumours of neural origin. Nevertheless, due to brain retraction and dissection, cerebral insult may occur during surgery. A recent study of patients who underwent meningioma resection demonstrated that pathologically increased serum S100β concentrations in the early postcraniotomy period correlated with neurological deterioration [11]. In this study, however, preoperative magnetic resonance imaging (MRI) parameters were not reported, tumours were not assessed volumetrically, and a very high rate of postoperative gross neurological deterioration occurred.

The current study was therefore conducted to examine the correlation between serial serum S100β protein levels and pre- and postcraniotomy MRI/computed tomography (CT) findings and neurological deterioration in patients undergoing meningioma resection. Revealing such associations would potentially promote the use of preoperative S100β level as a marker of tumour effect on brain tissue and postoperative S100β level as a marker for early detection of ongoing postcraniotomy brain damage.

## Materials and methods

### Patients

After institutional review board approval, all consecutive patients aged 18 to 80 years who were admitted to the Department of Neurosurgery for supratentorial meningioma surgery (Jan. 1 to Oct. 31, 2004) were prospectively screened for inclusion and informed consent was obtained. Excluded were patients who refused to participate or who had a history of chemotherapy/convulsions two weeks prior to admission, stroke/cardiopulmonary resuscitation/head trauma three months prior to admission, Alzheimer's disease, amyotrophic lateral sclerosis, prior melanoma, or brain neoplasm other than meningioma. Patients with chronic renal failure (creatinine >200 mmol/l) were also excluded due to potential interference with S100β clearance [12]. Patients were to be withdrawn from the study if they suffered an episode of hemodynamic instability (mean arterial pressure <60 mm Hg) which lasted more than 15 minutes and was non-responsive to fluid or vasopressor therapy at any time during the study period, regardless of cause. Occurrence of postoperative cerebral ischemia/hemorrhage was documented, but neither complication constituted a criterion for withdrawal.

### Perioperative management

Patients were enrolled upon admission on the day before surgery. Dexamethasone (≤16 mg/day) and phenytoin/valproic acid were prescribed individually. Anaesthesia was induced using thiopental or propofol, fentanyl, and vecuronium and was maintained with a balanced technique involving isoflurane, nitrous oxide, and oxygen. Additional doses of fentanyl were given at the anaesthesiologist's discretion. Ventilation was adjusted to maintain a PaCO_2 _(partial arterial pressure of carbon dioxide) of 30 to 35 mm Hg. Perioperative patient monitoring included intra-arterial blood-pressure monitoring. Surgery was performed by five neurosurgeons using standard techniques to minimise neural tissue damage. A neuronavigation system was used in convexity tumours to decrease the size of the craniotomy. In lesions in the base of skull, an extradural approach was opted for to reduce brain retraction. Extubation was performed in the operating room.

All patients were transferred postoperatively to the neurosurgical intensive care unit (ICU) for continued overnight monitoring. Further monitoring and treatment in the unit were provided at the discretion of the attending surgical ICU team, based on individual patient needs.

### Neurological evaluation

**Figure 1:**
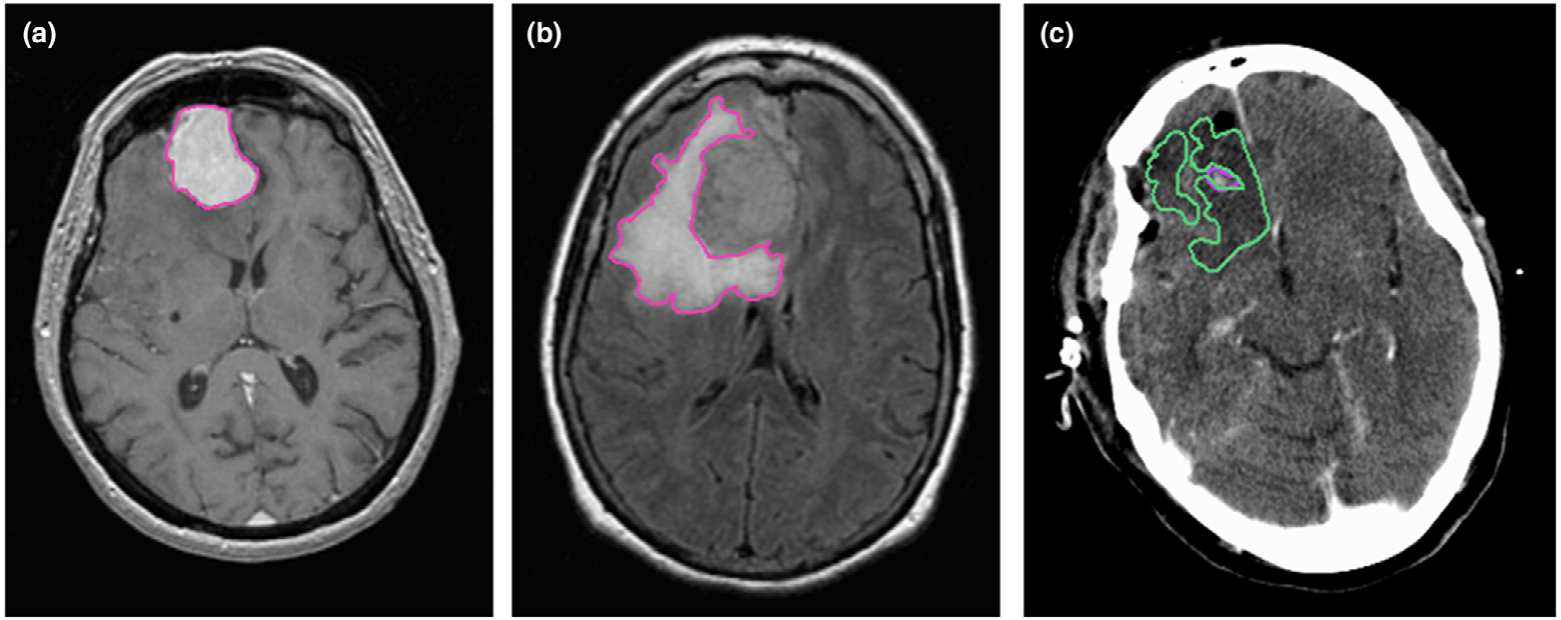
MRI/CT measurements of tumor and edema (a+b) and edema and hemorrhage (c) volumes.

Cranial nerve function and motor, sensory, language, and cerebellar function and a mini-mental state examination (MMSE) [13] were conducted preoperatively and 48 hours after surgery. All patients underwent MRI (T1, T2, T1 plus GAD [gadolinium] and FLAIR [fluid-attenuated inversion recovery] protocol) as part of their preoperative evaluation. CT scanning of the brain with and without contrast material was performed at 36 to 48 hours after surgery and repeated at the discretion of the treating physicians. All of the images were analysed by an independent team comprised of a neurosurgeon, radiologist, and physicist who were blinded to S100β levels and the study results. Tumour volume and brain edema were calculated using BrainSCAN™ software (ExacTrac^® ^computer technology; BrainLAB AG, Heimstetten, Germany), which is often used to plan radiosurgery treatment. For the purpose of the current study, the borders of the tumour and blood and/or edema were marked on each slice of the CT or MRI. This enables the program to construct a 3D model of the lesion area and measure its volume (Figure 1).

### S100β testing

Peripheral blood was sampled for S100β levels upon admission, immediately after surgery, and at 24 and 48 hours after surgery. All samples were centrifuged and stored (-70°C). Testing was performed using the Roche Elecsys^® ^S100 reagent kit (Roche Diagnostics GmbH, Penzberg, Germany) (assay duration 18 minutes, measuring range 0.005 to 39 pg/l, cross-reactivity against S100αα <1%). Less than 24 hours prior to testing, calibration was performed per reagent kit and control values were determined to be within the limits required for calibration (0.206 and 2.54 μg/l). The treating physicians were blinded to the results of the serum S100β tests.

### Data collection

Study data and medical records were collected prospectively, including patient demographics (for example, age, gender, and past medical history), neurological examination, intraoperative variables possibly related to surgical complexity (for example, duration of surgery and anaesthesia, surgical plane, resection grade, and blood loss), S100β levels, and relevant neurological tests.

No standard criteria were found in the literature for intraoperative definitions of the quality of the neurosurgical plane afforded by the tumour or its vascularity. A tumour presenting with a pial plane was therefore defined as a 'good' plane, and gross tumour invasion of the pia mater and the brain was defined as 'difficult' plane. It was assumed that dissection of the tumour from the brain would cause greater CNS tissue damage in the latter cases. The criteria for classification of the degree of tumour vascularity were arbitrary and based solely upon the senior neurosurgeon's assessment of the degree to which bleeding interfered with resection of the tumour.

### Endpoints

The study endpoints were determination of the relationship between preoperative serum S100β levels and MRI evidence of CNS damage and postoperative S100β levels and surgical complexity and postoperative clinical/radiological evidence of neural tissue injury.

### Statistical analysis

The study cohort included all patients who were enrolled into the study and who followed protocol procedures. First, the univariate results of all the research variables – predictors (independent variables) and outcome (the dependent variable) – were examined. Categorical variables (for example, patient gender, prior radiation/hormonal therapy, primary/recurrent disease, and medical history) are presented with their categories and the associated percentages. Numerical variables (for example, patient age and score in the MMSE) are presented with their means, standard deviations, medians, and ranges.

In the second step, the relationship between the outcome variables (serum S100β levels at each time point) and independent variables (variables potentially affecting these levels) was examined and their significance (*p *value) is presented. The Student *t *test, the Mann-Whitney test, and analysis of variance (ANOVA) were used to examine the relationship between categorical variables (dichotomous and multiple categories, respectively,) and S100β levels. Pearson and Kendall's tau-b correlations were used for the relationship between continuous variables and S100β levels measured in the various study time points (for example, preoperative tumour volume and baseline S100β levels, duration of surgery, and postoperative S100β levels). The results of the MMSE were analysed relative to the level of S100β as both a continuous variable and a dichotomous variable (deterioration versus non-deterioration) as was the presence/degree of midline shift on the postoperative CT scan. The statistical analyses were performed using SPSS 12 software (SPSS, Inc., Chicago, IL, USA).

## Results

### Patients, tumour pathology, and preoperative imaging

Fifty-six patients fulfilled entry criteria and were enrolled in the study. Three patients were excluded because they refused participation, and one was excluded because pathology disclosed hemangiopericytoma. Mean age was 58.5 ± 13 years, and 77% were female. Patient disease characteristics are presented in Table 1. All but one patient had a preoperative Glasgow Coma Score of 15. One patient had a score of 14 due to verbal deficit. Half of the patients scored maximal points in the preoperative MMSE. In the majority of patients (*n *= 34, 65%), pathological examination revealed transitional/meningothelial tumour, and in 48 patients (92%), the World Health Organization grading was 1 (Table 1). The average time from performance of the last preoperative MRI to surgery was 26.6 days. MRI demonstrated mass effect in 67% of the patients (Table 2).

### Surgery and outcome

Surgical data are presented in Table 3. The majority of surgical procedures (*n *= 45, 86%) were performed via pterional or frontal approaches. A neuronavigation system was used in 66% (21/32) of the operations that were not performed at the base of skull and in none (0/20) of the operations that were performed at the base of skull. The duration of surgery averaged 295 ± 154 minutes (Table 3).

Postoperative (48 hours after surgery) Glasgow Coma Scores remained 15 for all but three patients, who scored 14 (*n *= 2) and 10 (*n *= 1). Deterioration in motor performance, sensorium, and language skills occurred in 10, one, and two patients, respectively. MMSE scores decreased slightly from a mean preoperative score of 26.6 ± 6.8 to 26.0 ± 7.1 at the second postoperative day (*p *= not significant). Sixteen patients (31%) scored fewer points in the postoperative MMSE than in the preoperative MMSE.

Postoperative CT scan (Table 2) revealed evidence of blood in the surgical bed in 22 patients (42.3%) and brain edema in 35 patients (67%). The volume of bleeding was less than 1 cm^3 ^in 12 patients, 1 to 4 cm^3 ^in eight patients, and more than 4 cm^3 ^in two patients. The average edema volume was 19.28 ± 23.53 cm^3^. In one patient, brain infarction was found. Twelve patients (23%) had postoperative CT scan evidence of midline shift.

### Relationship between preoperative serum S100β levels and MRI evidence of CNS damage

Preoperative S100β levels did not correlate with tumour volume (*p *= 0.32), edema volume (*p *= 0.72), or tumour and edema volume together (*p *= 0.81) as measured by MRI. Other preoperative MRI variables such as presence of mass effect (ventricular asymmetry and/or midline shift), presence of homogenous enhancement, dural tail, or cystic component also did not correlate with preoperative serum S100β levels.

### Relationship between serum S100β levels and occurrence of neurosurgery

**Figure 2:**
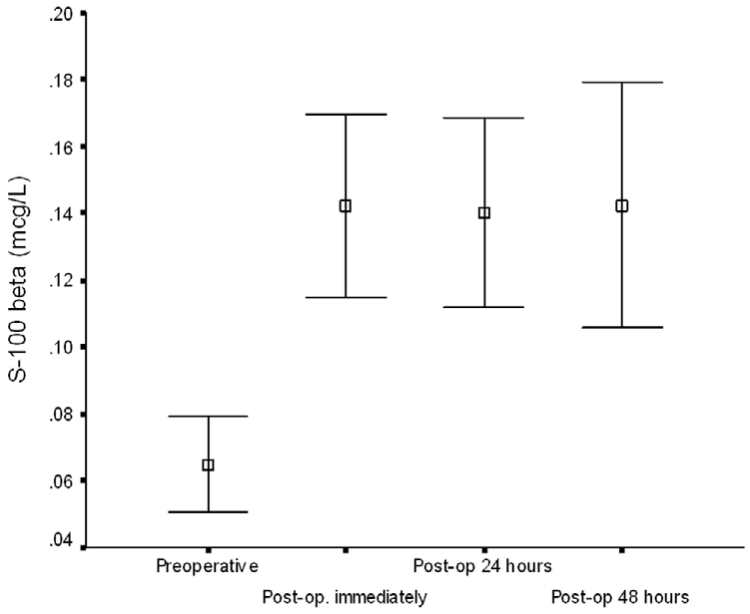
Serum S100β levels (average in micrograms per litre ± 95% confidence interval) at the various sampling times.

Initial serum S100β levels were 0.065 ± 0.040 μg/l (median 0.058 μg/l, range 0.009 to 0.204 μg/l) (Figure 2). These levels increased immediately postoperatively to 0.138 ± 0.081 μg/l (median 0.109 μg/l, range 0.022 to 0.313 μg/l) and remained elevated throughout the study period; at 24 hours, serum levels were maintained at 0.142 ± 0.084 μg/l (median 0.133 μg/l, range 0.043 to 0.498 μg/l) and at 48 hours at 0.155 ± 0.119 μg/l (median 0.126 μg/l, range 0.000 to 0.476 μg/l). Serum S100β levels were significantly different between pre- and all postoperative times (*p *< 0.0001) (Figure 2).

### Relationship between postoperative serum S100β levels and intraoperative variables

Serum S100β levels sampled 24 hours postoperatively correlated with the duration of surgery (*p *= 0.039). A difficult surgical plane (invasion of the pia mater and brain) was associated with higher immediate postoperative serum levels of S100β (0.189 ± 0.08 μg/l versus 0.121 ± 0.075 μg/l, *p *= 0.01, 95% CI 0.017 to 0.119), but no difference was observed at later sampling times (*p *= 0.102 at 24 hours and 0.198 at 48 hours).

S100β levels were compared for patients undergoing surgery with and without use of a neuronavigation system (regardless of surgical access route), and no difference was found between the two groups at any of the sampling times. Postoperative levels of S100β were also compared between surgery not performed in the base of skull (convexity, parasaggital, falx, and tentorial) and surgery performed in the base of skull (olfactory groove, tuberculum sella, and anterior clinoid), and no significant difference was found. ANOVA did not demonstrate a difference in S100β levels at any of the examined times between meningiomas with different degrees of vascularity (high, medium, and low).

### Relationship between postoperative S100β levels and postoperative evidence of neural tissue injury

#### Clinical examination

Deterioration in motor performance or decreased sensorium was not associated with higher postoperative elevations of serum S100β. Serum S100β levels were higher only 48 hours after surgery in patients with evidence of deterioration in language skills (no deterioration 0.147 ± 0.111 μg/l, deterioration 0.325 ± 0.205 μg/l [*p *= 0.037, 95% CI -0.344 to -0.012]). Overt clinical deterioration in neurological performance was associated with significantly higher serum S100β levels immediately after surgery (no deterioration 0.116 ± 0.071 μg/l, deterioration 0.168 ± 0.086 μg/l [*p *= 0.031, 95% CI -0.100 to -0.005]) but not later.

#### MMSE

**Figure 3:**
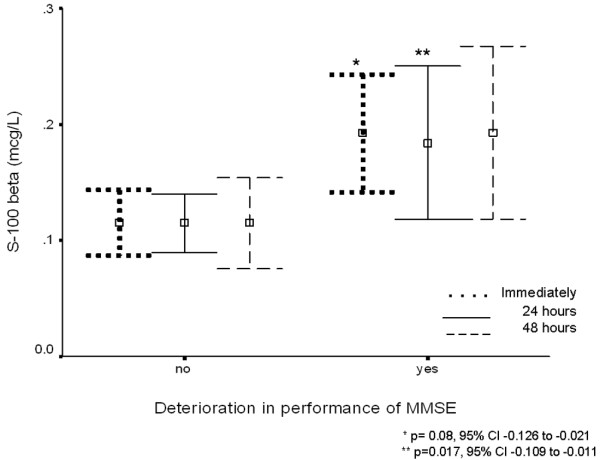
Postoperative serum S100β levels (average in micrograms per litre ± 95% confidence interval) with/without deterioration in mini-mental state examination (MMSE) performance.

The immediate and 24-hour postoperative S100β levels were higher among patients whose scores in the MMSE decreased postoperatively when compared with patients who retained or improved their capabilities (Figure 3). The severity of deterioration in the results of the MMSE correlated with the degree of elevation of postoperative serum S100β levels at all the sampling times (immediately [*p *= 0.005], 24 hours [*p *= 0.011], and 48 hours [*p *= 0.036] after surgery).

#### Radiology

**Figure 4:**
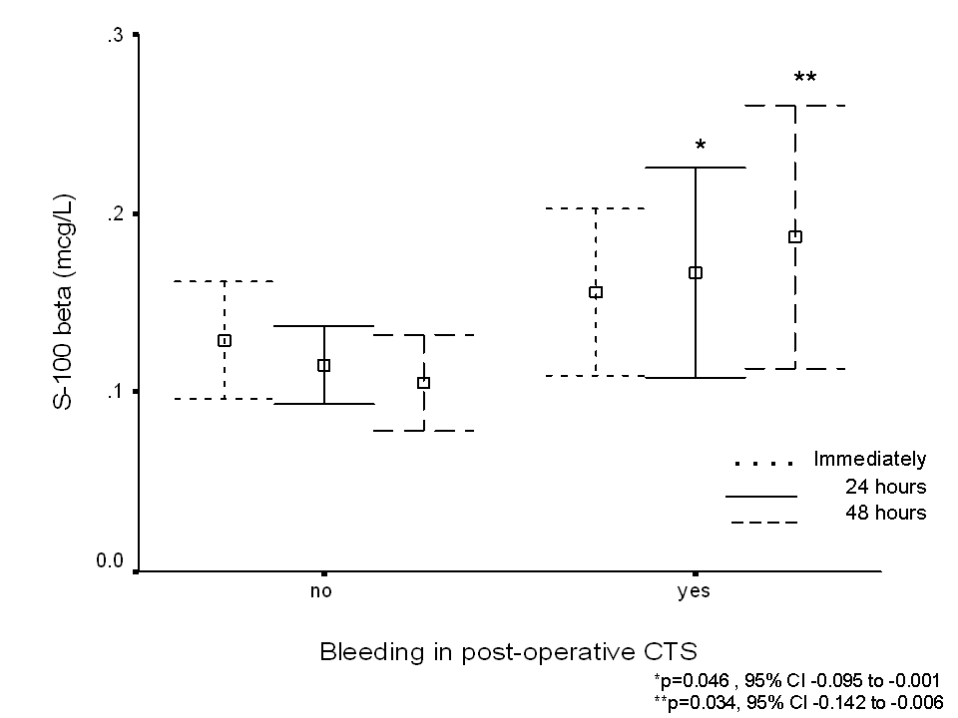
Serum S100β levels (average in micrograms per litre micrograms per litre is correct. ± 95% confidence interval [CI]) with/without postoperative computed tomography scan (CTS) evidence of bleeding.

Postoperative CT scan evidence of bleeding was associated with higher serum S100β levels at 24 and 48 hours after surgery (Figure 4) as was the presence of midline shift (0.126 ± 0.066 μg/l versus 0.206 ± 0.116 μg/l, *p *= 0.005, 95% CI -0.136 to -0.025 and 0.129 ± 0.104 μg/l versus 0.238 ± 0.130 μg/l, *p *= 0.006, 95% CI -0.186 to -0.032, respectively).

The degree of elevation in S100β levels also correlated with the severity of midline shift at these time points (*p *= 0.01 and 0.002, respectively). Edema was associated with higher serum S100β levels immediately after surgery (*p *= 0.022, 95% CI -0.092 to -0.007) and at 48 hours after surgery (*p *= 0.017, 95% CI -0.142 to -0.026). The severity of edema correlated with the degree of elevation of serum S100β levels at all sampling times (*p *= 0.05 immediately, *p *= 0.005 at 24 hours, and *p *< 0.001 at 48 hours after surgery). The serum S100β levels of the single patient who had postoperative CT scan evidence of infarction were significantly higher in the immediate postoperative (*p *= 0.019, 95% CI -0.342 to -0.034) and 24-hour (*p *= 0.009, 95% CI -0.3 to -0.048) postoperative measurements when compared with patients who had no CT scan evidence of bleeding or infarction.

## Discussion

This study is the first systematic examination of the relationship between serum S100β levels and MRI evidence of CNS damage caused by the presence of a homogenous group of extra-axial supratentorial tumours. It is also the first investigation of the link among postcraniotomy serum S100β levels, MMSE performance, and volumetric CT scan evidence of CNS compromise.

The data confirm that this marker performs poorly for characterisation and follow-up of this type of tumour. A correlation was found, however, between immediate postcraniotomy serum S100β levels and deterioration in performance of the MMSE. Persistently elevated early postoperative levels of serum S100β were also associated with postoperative CT scan evidence of bleeding, edema, or midline shift, suggesting a component of ongoing active release of S100β from glia accompanying secondary brain insult. This finding is of particular importance because the rise of this biomarker precedes clinical findings; patients are often incapable of undergoing complex clinical neurological testing at this stage.

Preoperative S100β levels did not correlate with either tumour and/or edema volume. It would be expected that a mass pressing the surrounding brain could have resulted in some S100β release. Plausible explanations for this finding include the slow lesion dynamics of this type of tumour; slow-growing tumours may cause less blood-brain barrier disruption, and the damage to the structural network supporting the neurons may be so gradual that even if S100β were released, current techniques would not be sensitive enough to distinguish the level between persons with no CNS lesion and those with a lesion causing a gradual mass effect. Molecular mechanisms for neuronal reorganisation (that is, plasticity) may also involve signaling cascades that affect astrocytes. Finally, S100β may have a role in processes occurring during acute but not chronic injury; it was recently demonstrated that S100β mRNA expression is potently downregulated after 12 and 24 hours of oxygen, serum, and glucose deprivation. Moreover, prolonged oxygen, serum, and glucose deprivation (for 48 hours)isassociated with a significant reduction of S100β release at later time intervals [14].

S100β levels were affected by the quality of surgical plane but not by the need to resect at a less-accessible tumour location (convexity located versus deep-seated). This may stem from the use of complex neurosurgical techniques intended to minimise retraction over the parenchyma of the brain (that is, extensive bone work, large craniotomies, extensive drilling of the skull base, and opening of the basal cisterns).

Our results are in agreement with previous work by Stranjalis *et al*. [11], who demonstrated in patients undergoing meningioma resection that the increased S100β level area under the curve up to seven days postcraniotomy was the most significant predictor for postoperative neurological deterioration and that those patients with increased postoperative S100β values had greater risk of poor outcome up to six months after surgery. In their study, however, preoperative MRI was not used, tumours were not assessed volumetrically, and a relatively high rate of gross neurological deterioration occurred postoperatively (immediate 50%, 6 months 30%).

In a study of operative decompression in patients suffering paresis due to metastatic spinal cord compression, functional outcome also correlated with S100β levels [10]. Patients with favourable outcomes had serum levels of S100β which were either normal all the time or increased initially but normalised within two to three days, whereas patients who had an unfavourable outcome also had continuously elevated levels (that is, levels either increased further or decreased slowly during 14 days). Elevated 10-day S100β levels were also predictive of the appearance of a new neurological deficit after surgery for aneurysmal subarachnoid hemorrhage [9]. The results from this study, in agreement with our study, confirmed that elevated serum S100β values after subarachnoid aneurismal hemorrhage correlate with postoperative CT scan findings such as infarction and vasospasm. Finally, serum S100β levels correlated with the size of the tumour-brain contact surface, which was closely related to the dimension of the surgical trauma, and with postoperative CNS damage caused by neurosurgical manipulation [15]. In the current study, use of neuronavigation did not moderate the increase observed in serum S100β levels but this finding may be related to surgical technique.

Correlations between deterioration in performance of the MMSE and elevations in S100β levels, similar to those found in the present study, have been reported after cardiopulmonary bypass surgery [2,3]. This has not been demonstrated after cardiac arrest and resuscitation [16], possibly due to the length of time that had elapsed between the event and MMSE testing or the small number of survivors available for testing.

There are several limitations to this study. Long-term follow-up to correlate serum S100β values with late outcome was not performed. One should also exercise caution in using serum S100β as the sole marker of brain damage; this protein may be released from injured tissues outside the brain, particularly from the heart, or mediastinum [17,18]. Experimental data in rat tissue found S100β in adipose, skin, and testicular tissue, albeit in significantly lower concentrations than in brain tissue [19]. Immunocytochemical approaches also demonstrated S100β in damaged skeletal muscle [20] and adipose tissue [21], and S100β protein has also been demonstrated in villous and intermediate trophoblast cells of the normal human placenta [22-24]. Rasmussen *et al*. [25] noted an increase in S100β 24 and 48 hours even after elective abdominal surgery and observed that this rise may be related to the appearance of postoperative delirium. S100β increases in the postoperative period may thus indicate more than one type of CNS derangement. S100β may also be involved in reparative processes after brain damage [26]. Sampling was limited to 48 hours after surgery although previous studies indicated ongoing damage for more than five days. Further sampling may have yielded additional information. Finally, in the current study, the S100β levels of patients who had postoperative CT scan evidence of bleeding overlapped with the levels of those who had none and postoperative edema was not consistently associated with elevated levels of S100β. Studies including larger sample populations are required to substantiate or disprove the relationship between these CNS pathologies and elevations in serum S100β levels.

## Conclusion

In patients with meningioma, serum S100β levels perform poorly as an indicator of tumour characteristics but may provide an early sign of postcraniotomy injury. Although the relevance of extracranial sources of S100β and the possible implications of participation of S100β in reparative processes after brain damage demand further investigation, the data suggesting that S100β protein does have potential as a prognostic clinical tool are increasing. The current study provides further substantiation to the mounting pool of data that serum S100β may be used as an early biomarker of acute neural tissue injury in the postoperative setting.

## Key messages

• Serum S100β levels perform poorly as an indicator of tumour characteristics for patients with meningiomas.

• Significant increases in serum S100β levels occur after neurosurgery prior to patient capability to undergo complex clinical neurological testing.

• Higher immediate postcraniotomy serum S100β levels correlate with eventual postoperative deterioration in performance of the MMSE.

• Persistently elevated postoperative levels of serum S100β (24 to 48 hours after craniotomy) are associated with postoperative CT scan evidence of bleeding, edema, and midline shift.

## Abbreviations

ANOVA = analysis of variance; CNS = central nervous system; CT = computed tomography; ICU = intensive care unit; MMSE = mini-mental state examination; MRI = magnetic resonance imaging.

## Competing interests

The authors declare that they have no competing interests.

## Authors' contributions

SE conceived, designed, and coordinated the study, analysed and interpreted the data, and drafted the manuscript. YS assisted in conceiving, designing, and coordinating the study. HO supervised the laboratory work and assisted in data interpretation and drafting of the manuscript. IM assisted in data interpretation and drafting of the manuscript. MH assisted in drafting of the manuscript. EI participated in study design, coordinated and participated in clinical data collection, carried out the laboratory work, and assisted in drafting of the manuscript. All authors read and approved the final manuscript.

## Figures and Tables

**Table 1:** Disease characteristics of the study population (*n *= 52)

Concomitant diseases		*n*	Percentage
Hypertension		16	31
Hyperlipidemia		15	29
Diabetes		8	15
Thyroid disorder		6	11
Other endocrine disorder		5	10
Chronic ischemic heart disease		5	10
Chronic obstructive pulmonary disease		5	10
Liver disease		3	6
Metabolic disorder		3	6
Meningioma			
Family history of meningioma		6	11
Prior irradiation		16	31
Prior hormonal therapy		5	10
Recurrent disease		14	27
Tumour histology	Transitional	22	42
	Meningothelial	12	23
	Fibrous	4	8
	Atypical	3	6
	Other^a^	11	21
WHO grading	1	48	92
	2	4	8

^a^Secretory, transitional and fibrous, meningiomatous, inflammatory, choroids, metaplastic, and psammomatous. WHO, World Health Organization.

**Table 2:** Radiological data: preoperative MRI and postoperative CT

Preoperative MRI	Tumour characteristic		*n*	Percentage
	Location	Convexity	10	19
		Parasaggital	19	36
		Tuberculum sella	9	17
		Anterior clinoid	5	10
		Olfactory groove	3	6
		Falx	3	6
		Sphenoid ridge	2	4
		Planum sphenoidale	1	2
	Mass effect	Ventricular asymmetry	23	44
		Midline shift	12	23
	Enhancement	Homogenous	43	83
		Cystic component	1	2
		Dural tail	13	25
	Bilateral		4	8
	Multifocal		4	8
		Mean	Median	Range
	Tumour volume (cm^3^)	35.29 ± 29.39	29.39	2.35 to 104.20
	Tumour edema-FLAIR (cm^3^)	24.83 ± 32.39	9.72	0 to 132.05
	Midline shift (mm)	2.16 ± 4.29	0	0 to 15.47
Postoperative CT scanning	Finding		*n*	Percentage
	Residual tumour		1	2
	Brain edema		35	67
	Brain infarct		1	2
	Midline shift		12	23
	Bleeding		22	42
		Mean	Median	Range
	Midline shift (mm)	1.29 ± 2.83	0	0 to 12.35
	Bleeding (cm^3^)	1.13 ± 4.19	0	0 to 29.68

CT, computed tomography; FLAIR, fluid-attenuated inversion recovery; MRI, magnetic resonance imaging.

**Table 3:** Surgical data

		*n*	Percentage
Approach	Pterional	24	46
	Frontal	21	40
	Parietal	7	14
Navigation	Yes	21	40
	No	31	60
Extent of excision (Simpson grade) [27]	1 – Macroscopically complete removal of dura, bone	39	75
	2 – Macroscopically complete removal, dural coagulation	11	21
	3 – Complete tumour resection, dura not coagulated	2	4
Plane	Difficult (invasion of the pia mater and brain)	14	27
	Good (pial plane)	38	73
Tumour vascularity	High	2	4
	Medium	29	56
	Low	21	40
	Mean ± SD	Median	Range
Length of anesthesia (minutes)	385 ± 159	355	195 to 870
Length of surgery (minutes)	295 ± 154	240	75 to 765
Blood loss (cc)	345 ± 189	300	50 to 1,000

SD, standard deviation.
